# Longitudinal Effects of Becoming a Family Caregiver: The Caregiving Transitions Study

**DOI:** 10.1093/geroni/igaa057.2271

**Published:** 2020-12-16

**Authors:** David Roth, Steven Zarit

## Abstract

Taking on caregiving responsibilities for older adult family members with disabilities is often considered to be a highly stressful experience that may adversely affect the health of caregivers. However, the vast majority of studies in this area compare existing samples of caregivers with questionably matched non-caregiving controls. In this symposium, we will present findings for a population-based sample of persons who became family caregivers while participating in a longitudinal epidemiological study. Changes in health and well-being are compared between these caregivers and non-caregiving control participants who were matched on multiple demographic and pre-caregiving health history variables. All persons enrolled as caregivers were providing sustained and substantial caregiving assistance. Presentations will include 1) a descriptive overview of the screening, eligibility, and enrollment methods used to construct these unique, population-based samples; 2) comparisons of within-person changes on measures of self-reported health and well-being for dementia and non-dementia caregivers; 3) changes in the caregivers’ social networks, social engagement, and leisure time activities; 4) comparisons of longitudinal changes on circulating inflammatory biomarkers (e.g., IL-6, CRP, TNF alpha receptor 1) and cellular aging (telomere length); and 5) examinations of individual differences in caregiver outcomes using a stress process model. Becoming a family caregiver can be stressful, but the opportunity to help a loved one and the related feelings of purpose and deepening family connections may also promote resilience and enhance health. These questions are far from resolved, and rigorous, prospective, population-based studies like the Caregiving Transitions Study promise to provide compelling new insights.

